# Editorial: Key proteins of tumor angiogenesis: potential therapeutic targets for gastrointestinal tumors

**DOI:** 10.3389/fmed.2024.1387567

**Published:** 2024-03-12

**Authors:** Weilong Zhong

**Affiliations:** Tianjin Key Laboratory of Digestive Diseases, Department of Gastroenterology and Hepatology, General Hospital, Tianjin Institute of Digestive Diseases, Tianjin Medical University, Tianjin, China

**Keywords:** gastrointestinal tumors, tumor metastasis, angiogenesis, drug targets, drug research

## Introduction

Tumor angiogenesis is a key process in tumor growth and metastasis, involving the formation of new blood vessels to supply the tumor with necessary oxygen and nutrients (1, 2). This process is particularly important in gastrointestinal tumors, as it promotes tumor growth and increases the likelihood of tumor metastasis through the circulatory system (3, 4). In recent years, with in-depth research into the tumor angiogenesis process, some key proteins have been found to play crucial roles (5). These proteins have become potential targets for gastrointestinal tumor therapy, offering possibilities for the development of new treatment methods (6, 7). However, despite the hope these therapeutic targets bring, many challenges remain in their clinical application (8). Therefore, accurately analyzing the mechanisms of gastrointestinal tumor development, developing and identifying new tumor therapeutic targets, and targeting either the tumor itself or its angiogenesis, while minimizing the impact on normal tissues, are the key research directions of focus in this topic.

Urbiola-Salvador et al., investigates the changes in plasma protein levels in colorectal cancer (CRC) patients, aiming to identify potential non-invasive biomarkers for early detection and prognosis. They focuses on colorectal cancer (CRC), exploring diagnostic tools, the significance of early detection, and the identification of potential blood-based biomarkers for CRC screening. Their research highlights the limitations of current screening methods and the promise of new biomarkers and technologies, such as the Proximity Extension Assay, for improving early detection and monitoring of CRC. Utilizing a proximity extension assay, the research quantified 690 proteins from plasma samples, finding 202 proteins significantly altered in CRC patients compared to healthy controls. Key findings include the identification of proteins related to Th17 activity, oncogenic pathways, and cancer-related inflammation, which could aid in CRC diagnosis. Specifically, proteins such as IFNG, IL32, and IL17C were associated with early CRC stages, while ACP6, FLT4, and MANSC1 correlated with advanced stages.

Fatemi et al.'s study, employing systematic review and meta-analysis methods, explored the association between polymorphisms in IGF1 pathway genes (IGF1, IGF1R, IRS1, and IRS2) and the risk of colorectal cancer (CRC) (Cheraghpour et al.). The study encompassed 26 research articles that met inclusion criteria, including 22,084 cases and 29,212 controls, aiming to fill the gap in the integration of genetic data related to the IGF pathway from previous studies. The IGF pathway plays a crucial role in the development and progression of CRC, and genetic variations may contribute to CRC risk. The scientific value of this study lies in its systematic analysis of multiple genetic variations in four genes within the IGF1 pathway, providing insights into the complex genetic mechanisms of CRC and valuable information for future research on CRC prevention and treatment. Moreover, considering the etiology of gastrointestinal tumors is closely related to the interaction between genetic and environmental factors, this study holds significant scientific value and clinical significance for revealing the link between specific genetic polymorphisms and CRC risk, thereby providing a theoretical basis for the early diagnosis and personalized treatment of gastrointestinal tumors.

Wang et al.'s article primarily investigated the association between serum gamma-glutamyl transferase (GGT) levels and high-grade colorectal adenomas. Through a retrospective study of 3,534 hospitalized patients, it was found that for every 20-unit increase in GGT levels, the risk of high-grade colorectal adenomas increased by 6%. Notably, individuals with GGT levels higher than 50 U/L had a 61% higher risk of high-grade colorectal adenomas compared to those with GGT levels below 50 U/L. This finding offers a new perspective for the early diagnosis of high-grade colorectal adenomas, potentially improving current screening strategies. The increase in GGT activity, an enzyme, may be related to tumor growth and development, especially in the colorectal adenoma-carcinoma sequence. Furthermore, the increase in GGT activity might promote tumor cells' resistance to chemotherapy drugs. The scientific value of this article lies in revealing the association between GGT and high-grade colorectal adenomas and in emphasizing the value of GGT as a potential diagnostic marker, providing new strategies for the early diagnosis and prevention of colorectal cancer. Additionally, this study opens new research directions for future studies on the diagnostic performance of GGT at different CRC stages, including high-grade colorectal adenoma stages.

Lin's work primarily investigated the role of the zinc transport pathway in colorectal cancer (CRC), particularly in colon adenocarcinoma (COAD), and its application in the construction of prognostic models (Chen et al.). The zinc transport pathway plays a key role in various tumors and may exert anti-tumor effects by improving immune function. The article discusses the importance of immunotherapy in the treatment of colorectal cancer, especially in metastatic CRC patients with mismatch repair deficiency and high microsatellite instability (dMMR-MSI-H). This study is closely related to the topic as it reveals the role of the zinc transport pathway in the development of colorectal cancer and provides possible targets for the development of new treatment strategies. By understanding the tumor's metabolic reprogramming and immune evasion mechanisms in depth, more precise treatment options can be provided for patients with colorectal cancer.

In summary, these studies provide us with a deep understanding of the mechanisms of gastrointestinal tumor development, especially colorectal cancer (CRC), and also reveal the important role of angiogenesis in tumor development. Through Víctor Urbiola-Salvador's study on plasma protein levels in CRC patients, we recognize Th17 activity, tumor-associated inflammatory pathways, and key proteins in tumor growth and development and identify specific proteins related to early and late stages of CRC, offering potential non-invasive biomarkers for early detection and prognosis assessment. Similarly, the studies by Fatemi et al. on the association between polymorphisms in IGF1 pathway genes and CRC risk, and by Wang et al. on the association between serum gamma-glutamyl transferase (GGT) levels and high-grade colorectal adenomas, further emphasize the importance of genetic and biochemical markers in early diagnosis and risk assessment. In particular, these study findings highlight the central role of angiogenesis and its regulatory mechanisms in the development of gastrointestinal tumors, including inflammatory responses within the tumor microenvironment, genetic variations, and interactions between tumor cells and host cells. Angiogenesis provides the tumor with essential nutrients and oxygen and participates in the dissemination and metastasis of tumor cells, transitioning the tumor from a local growth stage to an invasive and metastatic stage. Lin's research further reveals the role of the zinc transport pathway in tumor immune evasion and metabolic reprogramming, offering new targets for developing therapeutic strategies against the tumor microenvironment, especially angiogenesis. These findings help us better understand the mechanisms of gastrointestinal tumor development and provide new insights and tools for early diagnosis, therapeutic intervention, and prognosis assessment.

## Conclusion

In conclusion, by studying angiogenesis and its regulatory mechanisms in gastrointestinal tumors, we can better understand the complexity of tumor development and develop more effective diagnostic, therapeutic, and preventive strategies, thereby improving patients' survival rates and quality of life. Future research should continue to explore these potential biomarkers and therapeutic targets to promote innovation and improvement in gastrointestinal tumor management strategies.

## Author contributions

WZ: Writing—original draft.
